# An Association of Influenza Epidemics in Children With Mobile App Data: Population-Based Observational Study in Osaka, Japan

**DOI:** 10.2196/31131

**Published:** 2022-02-10

**Authors:** Yusuke Katayama, Kosuke Kiyohara, Tomoya Hirose, Kenichiro Ishida, Jotaro Tachino, Shunichiro Nakao, Tomohiro Noda, Masahiro Ojima, Takeyuki Kiguchi, Tasuku Matsuyama, Tetsuhisa Kitamura

**Affiliations:** 1 Department of Traumatology and Acute Critical Medicine Osaka University Graduate School of Medicine Suita Japan; 2 Department of Food Science Faculty of Home Economics Otsuma Women's University Tokyo Japan; 3 Department of Acute Medicine and Critical Care Center Osaka National Hospital National Hospital Organization Osaka Japan; 4 Department of Traumatology and Critical Care Medicine Osaka City University Graduate School of Medicine Osaka Japan; 5 Division of Trauma and Surgical Critical Care Osaka General Medical Center Osaka Japan; 6 Department of Emergency Medicine Kyoto Prefectural University of Medicine Kyoto Japan; 7 Division of Environmental Medicine and Population Sciences Department of Social and Environmental Medicine Osaka University Graduate School of Medicine Suita Japan

**Keywords:** syndromic surveillance, mobile app, influenza, epidemic, children

## Abstract

**Background:**

Early surveillance to prevent the spread of influenza is a major public health concern. If there is an association of influenza epidemics with mobile app data, it may be possible to forecast influenza earlier and more easily.

**Objective:**

We aimed to assess the relationship between seasonal influenza and the frequency of mobile app use among children in Osaka Prefecture, Japan.

**Methods:**

This was a retrospective observational study that was performed over a three-year period from January 2017 to December 2019. Using a linear regression model, we calculated the R^2^ value of the regression model to evaluate the relationship between the number of “fever” events selected in the mobile app and the number of influenza patients ≤14 years of age. We conducted three-fold cross-validation using data from two years as the training data set and the data of the remaining year as the test data set to evaluate the validity of the regression model. And we calculated Spearman correlation coefficients between the calculated number of influenza patients estimated using the regression model and the number of influenza patients, limited to the period from December to April when influenza is prevalent in Japan.

**Results:**

We included 29,392 mobile app users. The R^2^ value for the linear regression model was 0.944, and the adjusted R^2^ value was 0.915. The mean Spearman correlation coefficient for the three regression models was 0.804. During the influenza season (December–April), the Spearman correlation coefficient between the number of influenza patients and the calculated number estimated using the linear regression model was 0.946 (*P*<.001).

**Conclusions:**

In this study, the number of times that mobile apps were used was positively associated with the number of influenza patients. In particular, there was a good association of the number of influenza patients with the number of “fever” events selected in the mobile app during the influenza epidemic season.

## Introduction

Influenza can still spread in recent years and can cause death, especially in the elderly and infants. In addition, the spread of influenza not only harms people’s health but also has a significant social and economic impact due to absenteeism and missed work. Therefore, early surveillance to prevent the spread of influenza is a major public health concern. However, traditional infectious disease reports require 10 days to 2 weeks. Various early surveillance models using absenteeism records [1-3], pharmacy drug sales [4,5], and visits to emergency departments [6,7] have been studied. In recent years, there have also been early prediction models for influenza using Internet search engine data [3,8-12]. In Japan, based on the Infectious Disease Control Law, patients with influenza are reported by medical institutions to public health centers, and the public health departments of each prefecture announce the results. However, such traditional surveillance of infectious diseases is associated with high costs and considerable time from data collection to publication. Thus, we have previously revealed the relationship between telephone triage service data and the number of infectious disease patients in Osaka Prefecture, Japan [12,13].

In 2015, we developed a self-triage mobile app for the residents of Osaka Prefecture that determines the urgency of symptoms in children with sudden illness or injury and guides them to ambulances and appropriate medical institutions based on the urgency of their symptoms. The profile of users of this self-triage app has been revealed in detail [14]. This mobile app has been available for free download from Google Play and the App Store in Japan since 2016, and by the end of 2019, it had been downloaded 23,732 times and used a total of 63,230 times. If the relationship between the frequency of mobile app use and the number of influenza patients can be clarified and a prediction model can be structured, it may be possible to forecast influenza earlier and more easily. While traditional infectious disease reports actually require 10 days to 2 weeks, syndromic surveillance with mobile app data allows aggregation and forecasting to be done programmatically, enabling low-cost, real-time forecasting. The purpose of this study was to assess the relationship between seasonal influenza and the frequency of mobile app use among children in Osaka Prefecture, Japan.

## Methods

### Study Design, Population, and Setting

This was a retrospective observational study during a three-year period from January 2017 to December 2019. Osaka Prefecture has the largest urban area in western Japan, with an area of 1905.14 km^2^, a population of 8.8 million, and 1.09 million children of ≤14 years of age [15]. In this study, we included cases in which the child’s parents or guardians used the mobile app in Osaka Prefecture. Informed consent was obtained from the mobile app users at the time of mobile app use. This study was approved by the ethics committee of the Osaka University Graduate School of Medicine (approval no. 20313). This manuscript was written based on the STROBE statement (strengthening the reporting of observational studies in epidemiology) [16].

### Outpatient Surveillance of Influenza-Like Illness in Japan

The Infectious Disease Surveillance Program in Japan, initiated in 1981, formed the basis for influenza surveillance for outpatients [17,18]. This program was revised and updated to its present form following the revision of the Infectious Disease Control Law in 1999 [17-20]. The system is currently called the National Epidemiological Surveillance for Infectious Diseases, which includes a mandatory reporting system for nationally notifiable diseases and sentinel surveillance systems for various types of infectious diseases [21].

Influenza falls under the sentinel surveillance arm of the program. Weekly numbers of influenza patients have been reported to local health centers from 5000 medical institutions nationwide. Sentinel sites were designated according to their geographic distribution, type of medical institution (clinic or hospital), and population density. These sentinels use the following criteria for reporting influenza-like illness (ILI): (1) sudden onset of illness, (2) fever >38°C, (3) symptoms of upper respiratory inflammation, and (4) systemic symptoms such as general fatigue. A case is considered to meet the reporting criteria if the patient meets all symptoms from (1) to (4) or at least one of the symptoms in combination with a positive rapid diagnostic test [19]. Sentinel sites report the age group and sex of patients on a weekly basis. The report does not include personal information (eg, names or addresses). This information is transferred from local health centers to the prefectural government, where it is aggregated into a prefectural report. The report is then forwarded to the National Institute of Infectious Diseases in Tokyo, which is affiliated with the Ministry of Health, Labor and Welfare. Within Osaka Prefecture, 300 medical institutions report influenza patients to 10 local health centers [22]. In this study, the main endpoint was the weekly number of influenza patients in Osaka Prefecture. These data were acquired from the website of the Information Center of Infectious Diseases in Osaka Prefecture [22].

### Mobile App for Emergency Pediatric Patients

The details of the mobile self-triage app that was used in this study have been described previously [14]. First, the age and sex of the children were selected in this mobile app. Next, the user selects either “sickness” or “injury, poisoning, foreign substances, and others.” When either of these is selected, the list of chief complaints shown is displayed in the mobile app, and the user selects the relevant chief complaint. For example, if “fever” is selected, relevant signs and symptoms with high urgency, such as “fever of ≥41℃,” are displayed in the app. If none of these are selected, relevant signs and symptoms with moderate urgency, such as “decreased urine volume,” are displayed in the app. If none of them apply, the related signs and symptoms corresponding to “low urgency” are further displayed, and the urgency is determined based on the selected signs and symptoms. The app provides emergency medical services, such as the ability to call an ambulance or telephone triage center and information on available hospitals and clinics. Based on the judgment of urgency. If there is another chief complaint, such as “convulsion” when “fever” is selected, the app will move to the urgency assessment for the other complaint. Only hospitals and clinics in Osaka Prefecture that have agreed to register their information in the app will be displayed as available hospitals and clinics. In addition, the GPS feature of the user’s cellphone also provides a list of hospitals and clinics in order of proximity to the location where the app is being used. The Android version of this app was released in January 2016, while the iOS version was released in April 2016. The mobile app can be downloaded free from Google Play and the App Store in Japan.

### Endpoint

The endpoint of this study was the number of influenza patients≤14 years of age per week in the Osaka Prefecture. The number of influenza patients per week was obtained from data published on the website of the Osaka Institute of Public Health [22].

### Statistical Analysis

Using a linear regression model, we calculated the R^2^ value of the regression model to evaluate the relationship between the number of “fever” events selected in the mobile app and the number of influenza patients ≤14 years of age. We also calculated the Spearman correlation coefficient between the number of influenza patients and the calculated number of influenza patients ≤14 years of age, the adjusted R^2^ value, and the *P* value. The age groups were classified as follows: infants and toddlers (0-4 years), kids (5-9 years), and teenagers (10-14 years). The seasons were categorized as winter (January-March), spring (April-June), summer (July-September), and autumn (October-December). Then, we evaluated the interaction between influenza season (December-April) and the number of “fever” events selected in the mobile app. In addition, to assess the regression model in a smaller area, we divided Osaka Prefecture into eight regions (Toyono, Mishima, North-Kawachi, Middle-Kawachi, South-Kawachi, Sakai, Senshu, and Osaka City) (Multimedia Appendix 1). The eight regions were classified based on the medical care plan of the Osaka Prefectural Government [23]. Finally, we calculated Spearman correlation coefficients between the calculated number of influenza patients estimated using the regression model and the number of influenza patients, limited to the period from December to April, when influenza is prevalent in Japan. *P* values of <.05 were considered to indicate statistical significance. All statistical analyses were performed using SPSS version 23.0J (IBM Corp).

## Results

From 2017 to 2019, the mobile app was used a total of 59,375 times; 29,392 (49.5%) of these uses occurred in Osaka Prefecture. On the other hand, the number of influenza patients ≤14 years of age in the same period was 188,590 (Multimedia Appendix 2). Table 1 shows the characteristics of the subjects in this study. The median age was 1 year (IQR 0-3 years), and the most frequent age group was infants with 17,401 (59.2%) uses, followed by toddlers with 8999 (30.6%) uses. A total of 15,387 (52.4%) subjects were male, 13,788 (46.9%) were female, and 217 (0.7%) were of unknown sex. The mobile app was used 6453 (22.0%) times in 2017, 10,724 (36.5%) times in 2018, and 12,215 (41.6%) times in 2019. By season, the mobile app was used 6142 (20.9%) times in winter, 7714 (26.2%) times in spring, 7673 (26.1%) times in summer, and 7863 (26.8%) times in autumn. The region in which the app was most frequently used was Osaka City (13,570 times, 46.2%), and the region in which the app was used the least was the Mishima area (1156 times, 3.9%). The chief complaint most frequently selected on the mobile app was “fever” (14,777 times, 39.1%), followed by “cough” (2592 times, 6.9%) and “head and neck injury” (2523 times, 6.7%).

**Figure 1:**
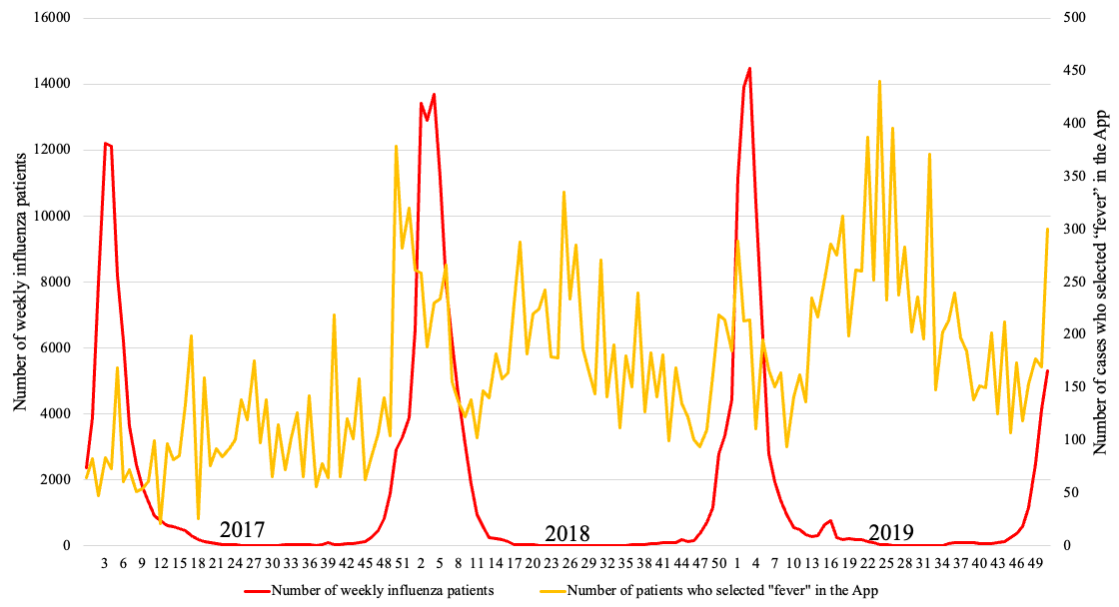
The weekly number of influenza patients and that of telephone triage counts due to "fever".

**Figure 2:**
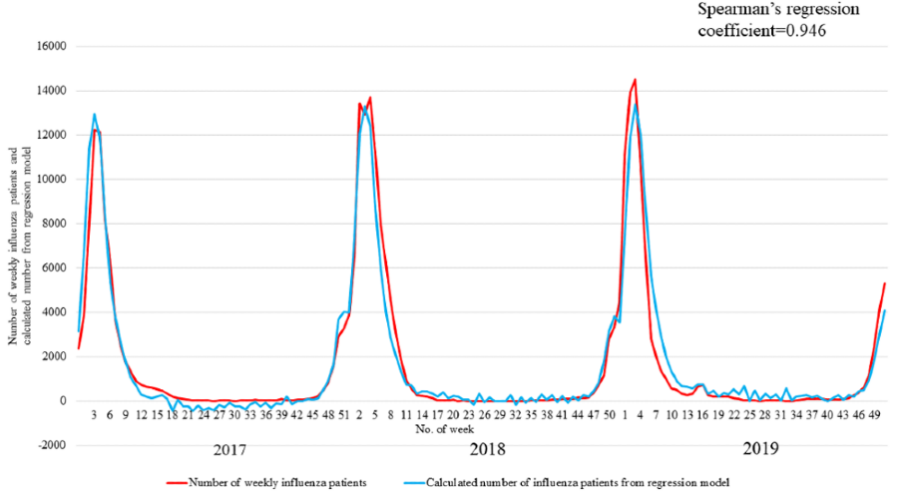
The weekly number of influenza patients and the calculated number of influenza patients from the linear regression model.

Figure 1 shows the weekly number of influenza patients ≤14 years of age and the weekly number of times that “fever” was selected in the mobile app. Figure 2 shows the weekly number of influenza patients ≤14 years of age and the calculated number of influenza patients ≤14 years of age estimated using the linear regression model. The red line shows the weekly number of influenza patients; the yellow line shows the number of times that “fever” was selected per week in the mobile app, and the blue line shows the calculated number of influenza patients ≤14 years of age estimated using the linear regression model. The regression coefficient of the weekly number of times that “fever” was selected in the mobile app was 2.977 (95% CI 0.680-5.314). The R^2^ for the linear regression model was 0.944, and the adjusted R^2^ value was 0.915. Spearman correlation coefficient between the number of influenza patients and the calculated number estimated using the regression model was 0.946. There was a significant interaction between influenza season and the number of times “fever” was selected in the mobile app (regression coefficient 5.873, 95% CI 2.521 to 9.226).

Table 2 shows the result of subgroup analysis by age group. The regression coefficient of the weekly number of times that “fever” was selected in the mobile app was 0.955 (95% CI –0.045 to 1.954) in infants and toddlers, 12.684 (95% CI 6.880 to 18.487) in kids, and 4.609 (95% CI –0.730 to 9.949) in teenagers, respectively.

**Table 1:** Demographic and clinical characteristics (N=29,392).

Characteristics			Values
**Age^a^ (years), n (%)**			
	Infants and toddlers (0-4)	25,279 (86.0)	
	Kids (5-9)	3032 (10.3)	
	Teenagers (10-14)	1081 (3.7)	
**Sex, n (%)**			
	Male	15,387 (52.4)	
	Female	13,788 (46.9)	
	Unknown	217 (0.7)	
**Time of telephone consultation and triage, n (%)**			
	Daytime (9:00 to 17:59)	11,818 (40.2)	
	Nighttime (18:00 to 8:59)	17,574 (59.8)	
**Year, n (%)**			
	2017	6453 (22.0)	
	2018	10,724 (36.5)	
	2019	12,215 (41.6)	
**Season, n (%)**			
	Winter (January to March)	6142 (20.9)	
	Spring (April to June)	7714 (26.2)	
	Summer (July to September)	7673 (26.1)	
	Autumn (October to December)	7863 (26.8)	
**Area, n (%)**			
	Osaka City Area	13,570 (46.2)	
	Middle-Kawachi Area	3071 (10.4)	
	Toyono Area	2893 (9.8)	
	Sakai Area	2546 (8.7)	
	Senshu Area	2315 (7.9)	
	North-Kawachi Area	2097 (7.1)	
	South-Kawachi Area	1744 (5.9)	
	Mishima Area	1156 (3.9)	
**Selected chief complaint in the mobile app, n (%)**			
	Fever	14,777 (39.1)	
	Cough	2592 (6.9)	
	Head and Neck Injury	2523 (6.7)	
	Nausea/Vomiting	2222 (5.9)	
	Convulsion	1635 (4.3)	
	Nasal Discharge	1571 (4.2)	
	Rash	1431 (3.8)	
	Diarrhea	1321 (3.5)	
	Face and extremities injury	995 (2.6)	
	Dyspnea	886 (2.3)	
	Other	7882 (20.8)	

^a^The median age was 1 year, with an IQR of 0 to 3 years.

**Table 2:** The subgroup analysis by each age group.

Regression model	Regression coefficient of the number of times “fever” is selected	95% CI
Infants and toddlers (0-4 years old)	0.955	–0.045 to 1.954
Kids (5-9 years old)	12.684	6.880 to 18.487
Teenagers (10-14 years old)	4.609	–0.730 to 9.949

**Figure 3:**
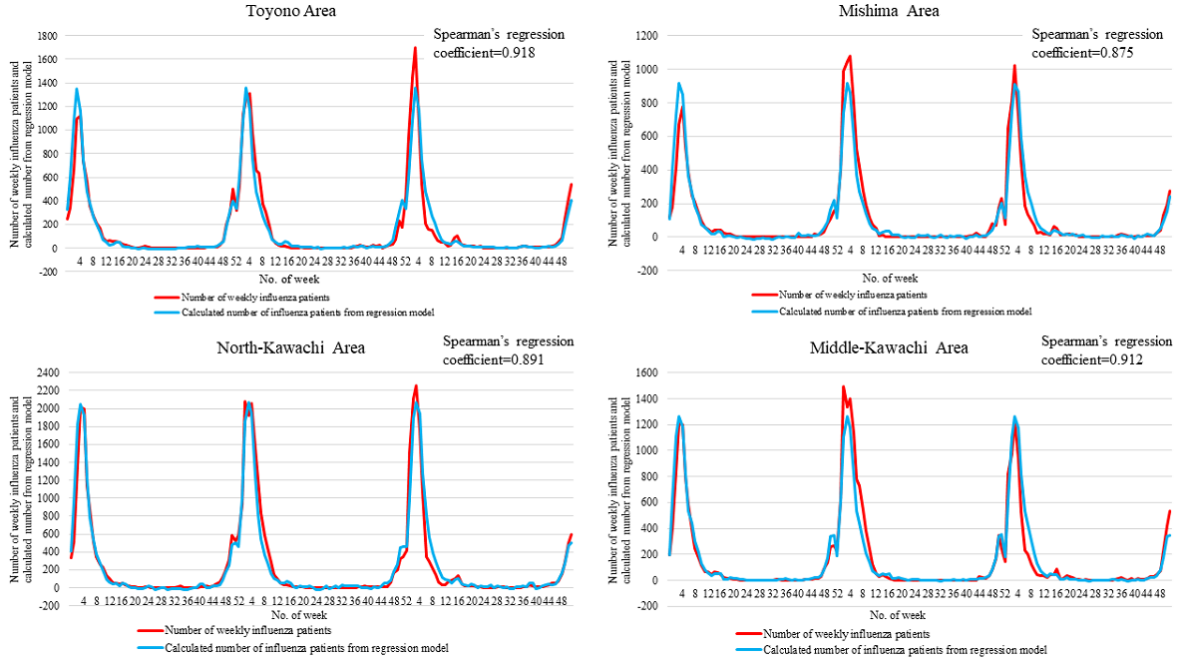
The weekly number of influenza patients and the calculated number of influenza patients from the linear regression model for each region.

**Figure 4:**
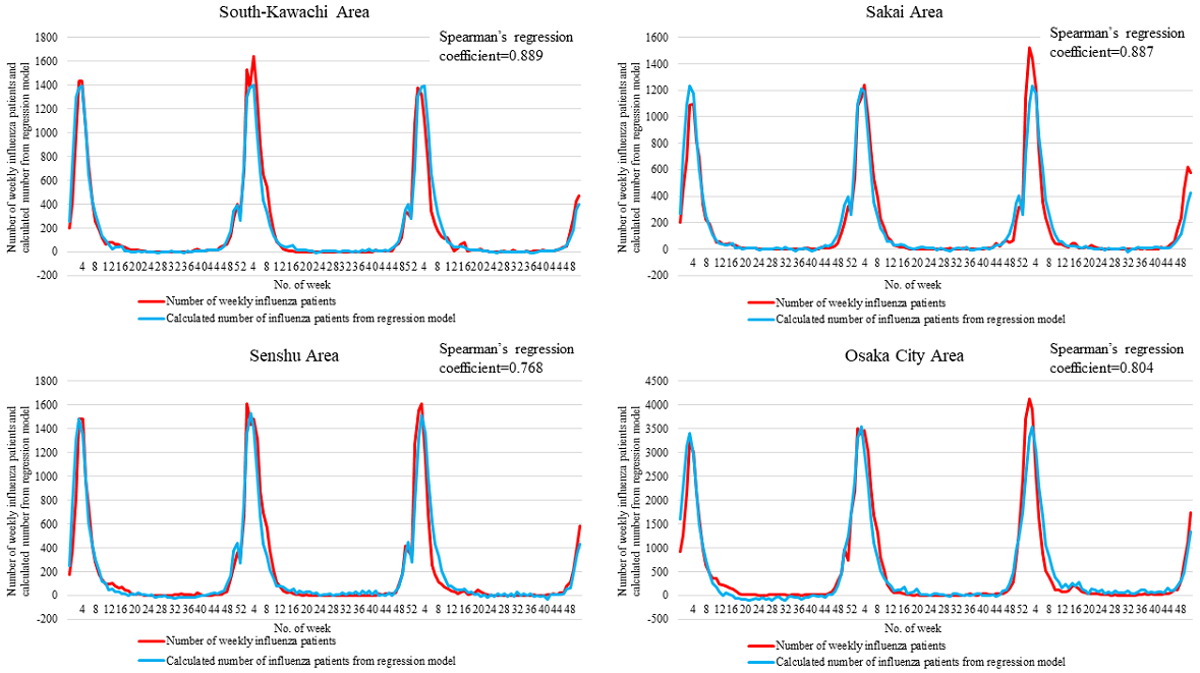
The weekly number of influenza patients and the calculated number of influenza patients from the linear regression model for each region.

Figures 3 and 4 show the number of influenza patients ≤14 years of age and the calculated number of patients estimated using the linear regression model for each region. The best correlation was observed in the Toyono area (Spearman correlation coefficient: 0.918; *P*<.001), and the worst correlation was observed in the Senshu area (Spearman correlation coefficient: 0.768; *P*<.001).

**Figure 5:**
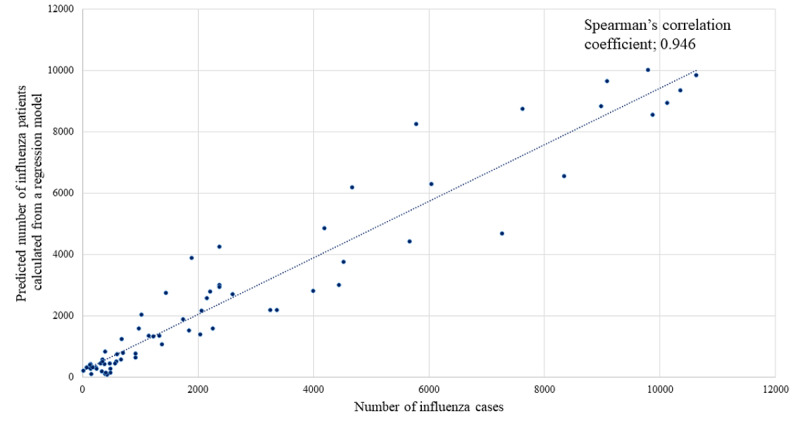
A scatter plot of the number of influenza patients and number of patients calculated using the liner regression model in December-April.

Figure 5 shows the relationship between the number of influenza patients ≤14 years of age and the calculated number estimated using the linear regression model during the influenza season (December–April). From December to April, the Spearman correlation coefficient between the number of influenza patients and the calculated number estimated using the linear regression model was 0.946 (*P*<.001).

## Discussion

### Principal Findings

This study revealed that the number of times that “fever” was selected in the mobile app was positively associated with the number of pediatric influenza patients in large urban areas of Japan. We also revealed that the validity of the linear regression model did not differ among the regions. In the analysis by age group, there was also no difference in the relationship between the number of times that “fever” was selected in the mobile app and the number of influenza cases. In the analysis by age group, there was also no difference in the relationship between the number of times that “fever” was selected in the mobile app and the number of influenza cases. The number of pediatric influenza patients and the calculated number from the linear regression model were highly correlated during the influenza epidemic periods (December–April) in Japan. Thus, the number of pediatric influenza patients was related to the number of times that the mobile app was used, and the contribution of the linear regression model was high. Although traditional infectious disease reports require 10 days to 2 weeks, there is a relationship between the number of times that the mobile app was used and the number of influenza patients during the influenza season, and comparing various models using this parameter will lead to the construction of an optimal prediction model in the future.

### Comparison With Prior Work

First, the number of pediatric influenza patients was strongly correlated with the calculated number estimated using the linear regression model based on the number of times that “fever” was selected in the mobile app. There have been several studies on syndromic surveillance of influenza using telephone triage data [24-26], and we also revealed that the number of calls for telephone triage was related to the number of influenza patients in Japan [13]. There have also been some syndromic surveillance studies of influenza using mobile app data. A study in Guatemala reported that the Pearson correlation coefficient between the number of ILI patients and the data of mobile apps that members of the general public used to record symptoms was –0.412 [27]. On the other hand, a study in South Korea reported that the Spearman correlation coefficient between the number of influenza patients and the data from a mobile app that supports parents and caregivers when young children have fever was 0.878 [28]. Spearman correlation coefficient between the number of influenza patients and the calculated number estimated using the regression model was 0.946 in this study. Although the number of participants was 189 and the maximum number of influenza patients was approximately 40 per week in the study by Guatemala. The number of participants in this study was approximately 15,000, while that in a study from South Korea was approximately 5 million. The number of infected patients and the data used in the analysis would affect the validity of the results. In order to accurately predict infectious disease epidemics, in addition to obtaining a large amount of data for the analysis, it is also necessary for the disease (eg, influenza) to infect a large number of people.

Next, we divided Osaka Prefecture into eight regions and analyzed the data, and found no difference in the validity of the linear regression model in any of the regions. These results were similar in the sub-group analysis divided by age group. Perry and colleagues compared the predictive performance of N4SID (a numerical algorithm for subspace state space system identification), exponentially weighted moving average, fast orthogonal search (FOS), and regression models in predicting the number of patients visiting the emergency department for respiratory diseases using the number of telephone consultations in Canada [7]. They found that the FOS model had better prediction accuracy than the regression model when the population was large but that the regression model had the best prediction performance in regions with small populations. In this study, we used a linear regression model to assess the relationship between the use of the mobile app and the number of influenza patients. The volume of data for which the linear regression model is suitable is not known; however, the fact that a good correlation was found between the number of influenza patients and the calculated number of influenza patients estimated using the regression model in all regions may indicate that this model was suitable for the analysis of the amount of data in this data set.

Third, the number of influenza patients and the calculated number of influenza patients estimated using the regression model were well correlated in the analysis of the influenza epidemic season from December to April. The same result was obtained in our previous study using telephone triage data [13]. In a previous study, the data set used in the regression model included the data from 17,000 individuals per year, while the number in this study was approximately 5000 per year. The relationship in the noninfluenza season was not as good as in the influenza season. In addition, there was an interaction between influenza season and the number of times “fever” was selected in the mobile app, and it is likely that the relationship is higher during the influenza season. This is because the number of times that “fever” was selected in the mobile app was used in this study. During the noninfluenza season, “fever” was selected due to other infectious diseases other than influenza, so the relationship between the number of pediatric influenza patients and the value calculated from the regression model might be low.

### Limitations

The present study was associated with some limitations. First, reports on influenza in Japan are fixed-point observations based on the Infectious Diseases Control Law, and the survey of influenza is not a survey of all cases. Second, regarding the criteria for reporting influenza patients in Japan, patients who are diagnosed with influenza based on clinical symptoms are included [29]. In Japan, it includes cases reported as influenza based on clinical symptoms alone without the use of an influenza diagnosis kit, so some of these patients may not truly have influenza. Third, as a result of the global spread of the novel COVID-19 in 2020, there has been a change in people’s attitudes toward the prevention of infectious diseases. Therefore, it may affect the frequency of mobile app usage and the number of influenza patients. Fourth, since this study included pediatric patients, it is unclear whether it is appropriate for predicting epidemics in adult and elderly populations. Fifth, as this study followed the assumptions of linear regression analysis and it was a population-based study in a metropolitan area of Japan, the generalizability of these results may be high. However, it has not been assessed for validity in other regions, so external validity needs to be assessed, and we will assess external validity in the future.

### Conclusions

In this study, the number of times that mobile apps were used was positively associated with the number of influenza patients. In particular, the relationship between the number of times that mobile apps were used and the number of influenza patients was good during the influenza epidemic season.

## Appendix

Multimedia Appendix 1 Map of medical districts in Osaka prefecture.

Multimedia Appendix 2 The weekly number of times that "fever" was selected in the mobile app and the weekly number of influenza patients in Osaka, Japan between 2017 and 2019.

## Abbreviations

FOS: fast orthogonal search
ILI: influenza-like illness

## References

[1] Kara EO, Elliot AJ, Bagnall H, Foord DGF, Pnaiser R, Osman H, Smith GE, Olowokure B (2011). Absenteeism in schools during the 2009 influenza A(H1N1) pandemic: a useful tool for early detection of influenza activity in the community?. Epidemiol Infect.

[2] Fan Y, Yang M, Jiang H, Wang Y, Yang W, Zhang Z, Yan W, Diwan VK, Xu B, Dong H, Palm L, Liu L, Nie S (2014). Estimating the effectiveness of early control measures through school absenteeism surveillance in observed outbreaks at rural schools in Hubei, China. PLoS One.

[3] Ma T, Englund H, Bjelkmar P, Wallensten A, Hulth A (2014). Syndromic surveillance of influenza activity in Sweden: an evaluation of three tools. Epidemiol Infect.

[4] Vergu E, Grais RF, Sarter H, Fagot J, Lambert B, Valleron A, Flahault A (2006). Medication sales and syndromic surveillance, France. Emerg Infect Dis.

[5] Socan Maja, Erculj Vanja, Lajovic J (2012). Early detection of influenza like illness through medication sales. Cent Eur J Public Health.

[6] Hall G, Krahn T, Van Dijk A, Evans G, Moore K, Maier A, Majury A (2013). Emergency department surveillance as a proxy for the prediction of circulating respiratory viral disease in Eastern Ontario. Can J Infect Dis Med Microbiol.

[7] Perry AG, Moore KM, Levesque LE, Pickett CWL, Korenberg MJ (2010). A Comparison of Methods for Forecasting Emergency Department Visits for Respiratory Illness Using Telehealth Ontario Calls. Can J Public Health.

[8] Hulth A, Rydevik G, Linde A (2009). Web queries as a source for syndromic surveillance. PLoS One.

[9] Ginsberg J, Mohebbi MH, Patel RS, Brammer L, Smolinski MS, Brilliant L (2009). Detecting influenza epidemics using search engine query data. Nature.

[10] Davidson MW, Haim DA, Radin JM (2015). Using networks to combine "big data" and traditional surveillance to improve influenza predictions. Sci Rep.

[11] Dong X, Boulton ML, Carlson B, Montgomery JP, Wells EV (2017). Syndromic surveillance for influenza in Tianjin, China: 2013-14. J Public Health (Oxf).

[12] Katayama Y, Kiyohara K, Komukai S, Kitamura T, Ishida K, Hirose T, Matsuyama T, Kiguchi T, Shimazu T (2021). Relationship between the number of pediatric patients with rotavirus and telephone triage for associated symptoms. Am J Emerg Med.

[13] Katayama Y, Kiyohara K, Komukai S, Kitamura T, Ishida K, Hirose T, Matsuyama T, Kiguchi T, Hirayama A, Shimazu T (2020). The relationship between seasonal influenza and telephone triage for fever: A population-based study in Osaka, Japan. PLoS One.

[14] Katayama Y, Kiyohara K, Hirose T, Matsuyama T, Ishida T, Nakao S, Tachino Jotaro, Ojima Masahiro, Noda Tomohiro, Kiguchi Takeyuki, Hayashida Sumito, Kitamura Tetsuhisa, Mizobata Yasumitsu, Shimazu Takeshi (2021). A Mobile App for Self-Triage for Pediatric Emergency Patients in Japan: 4 Year Descriptive Epidemiological Study. JMIR Pediatr Parent.

[15] The Census in Osaka Prefecture in 2015. Osaka Prefectural Government.

[16] von Elm E, Altman DG, Egger M, Pocock SJ, Gøtzsche PC, Vandenbroucke JP (2007). The Strengthening the Reporting of Observational Studies in Epidemiology (STROBE) statement: guidelines for reporting observational studies. The Lancet.

[17] Nakamura Y, Sugawara T, Kawanohara H, Ohkusa Y, Kamei M, Oishi K (2015). Evaluation of Estimated Number of Influenza Patients from National Sentinel Surveillance Using the National Database of Electronic Medical Claims. Jpn J Infect Dis.

[18] Murakami Y, Hashimoto S, Kawado M, Ohta A, Taniguchi K, Sunagawa T, Matsui T, Nagai M (2016). Estimated Number of Patients with Influenza A(H1)pdm09, or Other Viral Types, from 2010 to 2014 in Japan. PLoS One.

[19] Okabe N, Yamashita K, Taniguchi K, Inouye S (2000). Influenza surveillance system of Japan and acute encephalitis and encephalopathy in the influenza season. Pediatr Int.

[20] Shimada T, Sunagawa T, Taniguchi K, Yahata Y, Kamiya H, Yamamoto KU, Yasui Y, Okabe N (2015). Jpn J Infect Dis.

[21] Infectious Disease Surveillance System in Japan. National institute of infectious diseases.

[22] The Information Center of Infectious Disease in Osaka Prefecture.

[23] The Health and Medical Care Plan by Osaka Prefectural Government. Osaka Prefectural Government.

[24] Lucero-Obusan C, Winston CA, Schirmer PL, Oda G, Holodniy M (2017). Enhanced Influenza Surveillance Using Telephone Triage and Electronic Syndromic Surveillance in the Department of Veterans Affairs, 2011-2015. Public Health Rep.

[25] Yih WK, Teates KS, Abrams A, Kleinman K, Kulldorff M, Pinner R, Harmon R, Wang S, Platt R (2009). Telephone triage service data for detection of influenza-like illness. PLoS One.

[26] Moore K (2004). Real-time syndrome surveillance in Ontario, Canada: the potential use of emergency departments and Telehealth. Eur J Emerg Med.

[27] Prieto JT, Jara JH, Alvis JP, Furlan LR, Murray CT, Garcia J, Benghozi P, Kaydos-Daniels SC (2017). Will Participatory Syndromic Surveillance Work in Latin America? Piloting a Mobile Approach to Crowdsource Influenza-Like Illness Data in Guatemala. JMIR Public Health Surveill.

[28] Kim M, Yune S, Chang S, Jung Y, Sa SO, Han HW (2019). The Fever Coach Mobile App for Participatory Influenza Surveillance in Children: Usability Study. JMIR Mhealth Uhealth.

[29] Zaraket H, Saito R (2016). Japanese Surveillance Systems and Treatment for Influenza. Curr Treat Options Infect Dis.

