# Supplementary figures and images for: An Association of Influenza Epidemics in Children With Mobile App Data: Population-Based Observational Study in Osaka, Japan
Source: JMIR Form Res. 2022 Feb 10;6(2):e31131. doi: 10.2196/31131 (PMC8874815; doi:10.2196/31131)

## Supplemental Figure

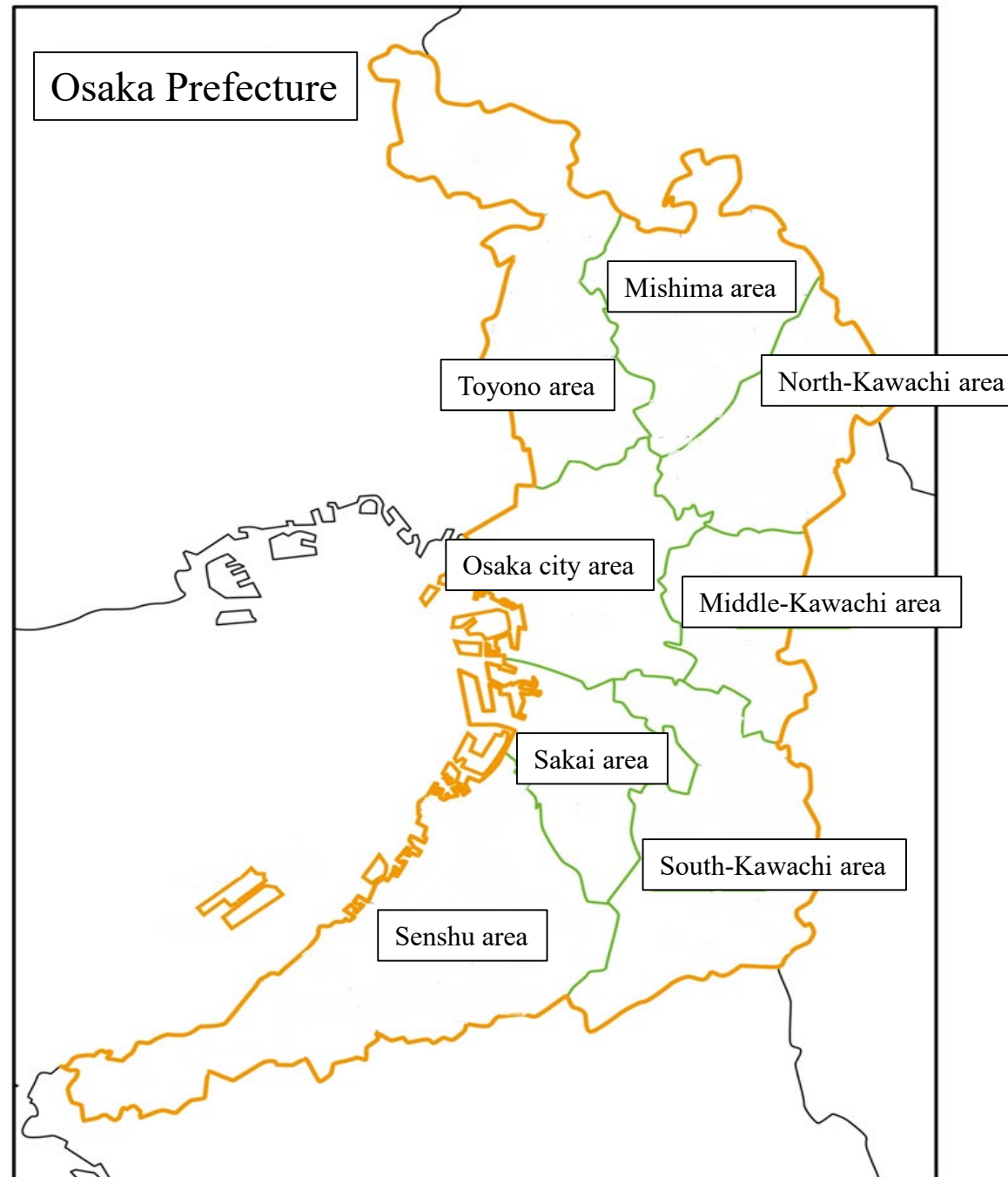

Supplement: Multimedia Appendix 1 [file formative_v6i2e31131_app1.pdf]
